# A New and Integral Approach to the Etiopathogenesis and Treatment of Breast Cancer Based upon Its Hydrogen Ion Dynamics

**DOI:** 10.3390/ijms21031110

**Published:** 2020-02-07

**Authors:** Salvador Harguindey, Khalid Alfarouk, Julián Polo Orozco, Kévin Hardonnière, Daniel Stanciu, Stefano Fais, Jesús Devesa

**Affiliations:** 1 Institute of Clinical Biology and Metabolism, Postas 13, 01004 Vitoria, Spain; polorozco@gmail.com; 2 Al-Ghad International Colleges for Applied Medical Sciences, Al-Madinah Al-Munawarah, Saudi Arabia and Alfarouk Biomedical Research LLC, Tampa, FL 33617, USA; khaliomer@gmail.com; 3 Université Paris-Saclay, Inserm, Inflammation, Microbiome and Immunosurveillance, 92290 Châtenay-Malabry, France; kevin.hardonniere@u-psud.fr; 4 Scientific Direction, MCS Foundation For Life, 5623KR Eindhoven, The Netherlands; cd.stanciu@gmail.com; 5 Department of Oncology and Molecular Medicine, Istituto Superiore di Sanità (National Institute of Health), Viale Regina Elena, 299, 00161 Rome, Italy; Stefano.fais@iss.it; 6 Scientific Direction, Foltra Medical Centre, Travesía de Montouto 24, 15886 Teo, Spain; devesa.jesus@gmail.com

**Keywords:** pH and breast cancer, breast cancer etiology, breast cancer pathogenesis, breast cancer treatment, pH-centric anticancer paradigm, hydrogen ion dynamics of cancer, cancer proton reversal, multiple drug resistance (MDR) integral approach

## Abstract

Despite all efforts, the treatment of breast cancer (BC) cannot be considered to be a success story. The advances in surgery, chemotherapy and radiotherapy have not been sufficient at all. Indeed, the accumulated experience clearly indicates that new perspectives and non-main stream approaches are needed to better characterize the etiopathogenesis and treatment of this disease. This contribution deals with how the new pH-centric anticancer paradigm plays a fundamental role in reaching a more integral understanding of the etiology, pathogenesis, and treatment of this multifactorial disease. For the first time, the armamentarium available for the treatment of the different types and phases of BC is approached here from a Unitarian perspective-based upon the hydrogen ion dynamics of cancer. The wide-ranged pH-related molecular, biochemical and metabolic model is able to embrace most of the fields and subfields of breast cancer etiopathogenesis and treatment. This single and integrated approach allows advancing towards a unidirectional, concerted and synergistic program of treatment. Further efforts in this line are likely to first improve the therapeutics of each subtype of this tumor and every individual patient in every phase of the disease.

## 1. Introduction

According to The International Agency For Research on Cancer (IARC), breast cancer (BC) is the most common malignant tumor in humans and the secondary cause of mortality of cancer in women, just behind lung cancer [1]. BC-related morbidity is primarily due to a progressive metastatic process [2]. Many associated risk factors, either genetic, from *BRCA1* and *BRCA2* gene mutations, a wide array of other genetic derangements [3], and a multiplicity of environmental factors such as age, obesity and estrogens, among many others [4], are involved in the onset of BC. Despite significant advances in therapy, the overall results are not too successful, especially in advanced disease [5,6]. This may indicate that a change towards a more comprehensive and perhaps radically different perspective is necessary in order to incorporate more rational and less toxic treatments, and at the same time foster a better understanding of this multifaceted disease.

A new and all-comprehensive pH-related paradigm has increasingly grown during the last few years, notably after the realization that the cancer-selective intracellular/extracellular pH deregulation is critical in the control of many cellular, both normal and pathological, processes [7]. One of the latest achievements of the new pH-paradigm has been to describe the integral and unitarian mechanism mediating the wide array of apparently unrelated factors involved in the etiopathogenesis of cancer, a finding that we can now be applied to BC [8].

The study of the abnormal hydrogen ion (H^+^) dynamics of cancer started almost five decades ago [9,10,11,12,13]. Since then, a rapid increase in the understanding of the deregulated H^+^ dynamics in cancer and the cancer-specific proton gradient reversal (CPR) has resulted in a new and increasingly outreaching paradigm, known as the pH-centric anticancer paradigm. This perspective embraces many different aspects of basic, preclinical and clinical oncology, all derived from this H^+^-related energetic concept that has allowed an intimate acid-base approach to the inner nature of malignancy. Nowadays the pH or H^+^- related model is already able to unite different fields, from molecular biology to biochemistry and the metabolism of cancer, having already reached up to the clinical aspects of cancer cells and tissues [14,15]. This perspective has rapidly extended to other different collateral areas of oncological research, incorporating within its range areas previously far apart when approached by the old and reductionist model, such as angiogenesis, environmental carcinogenesis and cancer immunology, also including the initiation, progression, metastatic process and even the spontaneous regression of cancer [16,17,18,19,20,21]. In summary, this integral and comprehensive paradigm can embrace most, if not all, aspects of cancer, and also of BC, from etiopathogenesis to treatment.

The cancer-selective abnormalities of intracellular alkalization plus extracellular acidification of all types of solid tumors and leukemias (CPR) represent the mirror image of normality, an upside-down disruption and tip over from normal homeostasis and allostasis [8]. Normality is exactly the opposite: a more acidic medium inside than outside non-cancerous cells [22,23]. As a consequence of this acid-base homeostatic disruption and energetic failure of cellular hydrogen ion (H^+^) dynamics, attempts to induce intracellular acidification using proton transport and pump inhibitors (PTIs and PPIs), as well as other intracellular acidifiers of different origins and natures (repurposed drugs), has become a new and valuable therapeutic strategy in selective cancer treatment. In this vein, the wide-ranging applications and potential benefits of this approach to the therapeutics of solid tumors has been recently published in a full issue containing fourteen reviews addressing the different aspects of the new pH-centric anticancer paradigm [24].

In the same line, we recently published an original review dealing with the pH-related possibilities in the treatment of brain malignancies in humans [25]. To uncover further pathophysiological and therapeutic applications of this post-traditional and non-main stream approach to cancer, this basic to clinically oriented and translational review will also discuss the foundations of the H^+^-related paradigm now applied in this contribution to the etiopathogenesis and treatment of BC [8].

## 2. Cancer as an Acid-Base Disease

The highest and lowest limits of pH in cellular and human life are considered to range between 6.8 and 7.8, a difference of only one unit. The normal cellular pH (pHi) is accepted to be 7.0–7.1, while a normal interstitial and systemic pH (pHe) is considered to be 7.35–7.45. Otherwise, the pHi of malignant cells can be as high as 7.8 [8]. Contrariwise, during malignant cells apoptosis the pHi has been shown to be as low as to 5.0 [26]. Most importantly, seminal studies in this area demonstrated that malignant transformation takes place at a pHi > 7.2, that is, only 0.2 units above normality, the time period needed for the oncogenic effect of the acid-base change being inversely related to the pHi increase [27].

During the last few years the new pH-centric anticancer paradigm and the H^+^ dynamics of cancer have helped to significantly increase the understanding of the intimate nature of human malignant tumors. Nowadays, it is agreed that all cancer cells and tissues have deeply rooted evolutionary and thermodynamic pH-related advantages over all normal tissues. This characteristic allows neoplastic cells first to survive in the most hostile conditions, then to grow locally, later to invade neighboring tissues to finally disseminate out of control, overwhelming all the defensive barriers and immune mechanisms of the host: “the neostrategy of cancer cells and tissues”. These highly pathological energetic and metabolic disruptions are based on a pathognomonic intracellular alkalization of cancer cells and, secondarily, to an extracellular and intratumoral microenvironmental acidosis. Both coordinated phenomena are facilitated by overactive membrane-bound proton transporters (PT) and pumps (PP) extruding mechanisms, inducing an inversion of the normal cells/surrounding tissues pH gradient (pHi to pHe), or CPR, across cellular membranes. These deregulated pH dynamics also determine the cancerous effects on normal cells and tissues, from early tumorigenesis and transformation to proliferation, local growth and a metastatic process that usually ends up with the death of the host.

Such metabolic reprogramming confers to cancer cells and tissues other important thermodynamic advantages, such as enhancing their resistance to hypoxia and to cancer therapy (MDR), allowing them to survive under almost any conditions. Finally, these dynamic changes allow malignant cells and tissues to avoid the pro-apoptotic intracellular acidification (IA), which would result in a selective cancer cell death as the successful outcome of treatment. As previously mentioned, the new acid-base approach to cancer has extended side-ways to the point that it can now provide further meaning to most, if not all, the hallmarks previously described for cancer, and even envision new ones [28,29]. Such a conceptual deepening into the intimate nature of malignancy allows the new H^+^-related paradigm to cover under one single heading the many different areas and hallmarks of cancer research and treatment previously disconnected to a large extent, namely:

(a) pH and the Warburg effect: Recent publications of our group and others have defended that the Warburg effect can be fully explained by the selective pH abnormalities of cancer cells and their effect on aerobic glycolysis [8,24,25,30,31,32,33,34,35,36].
(b) pH abnormalities in the etiology and pathogenesis of BC and other tumors: Nowadays, intracellular (IC) alkalization is increasingly recognized as a fundamental and sine qua non factor in cellular transformation in BC and other malignant tumors [8,37,38,39,40,41,42,43,44].
(c) pH and cancer molecular biology, biochemistry and metabolism: Recent experimental data have clearly shown that the pH-related paradigm can reinterpret the molecular biology, biochemistry and intermediary metabolism of cancer cells and tissues from an integral and Unitarian dynamic perspective [8,37,38,39,40,41,42,45,46].
(d) pH, tumor growth and invadopodia: Local invasion is promoted by Na^+^/H^+^ mediated low pH of invadopodia at the advancing edge of cancer cells [37,47,48].
(e) pH and microenvironmental-intratumoral acidosis in cancer growth and dissemination: Through different mechanisms, from biochemical to immunological, the high extrusion of lactic acid and other metabolites from cancer cells creates a highly acidified extracellular media that stimulates different and coordinated mechanisms strategically organized to favor local growth, invasion and dissemination [8,22,49,50,51,52,53,54].
(f) pH and the metastatic process: The pHi/pHe gradient reversal (CPR) is fundamental in all stages of cancer growth, from cell transformation and the initiation of tumor growth to the activity and progression of the metastatic process [23,35,48,55].
(g) pH, proton transporters (PTs), proton pumps (PPs) and their inhibitors (PTIs and PPIs): During the last decade the increasing interest and knowledge of the different membrane-bound PTs and PPs in cancer pathogenesis, as well as their inhibitors in cancer treatment, has made it possible to approach, from a different and non-mainstream perspective, the latest therapeutic efforts in cancer treatment, either in BC or in other human malignant tumors [15,36,40,42,54,55,56,57,58,59,60,61,62,63,64,65,66,67,68,69,70].
(h) pHi acidification and reverting cancer proton reversal (CPR) in cancer treatment: Attempts to revert CPR in cancer are the fundamental therapeutic issue in the entire paradigm of the H^+^-related dynamics of cancer, BC included, from the initiation stage to angiogenesis and to the treatment of metastatic disease [8,16,23,55,63,71].
(i) pH and voltage-gated sodium channels (VGSC): The expression of Na+ channels synergically associated with Na^+^/H^+^ antiporter activity and over-expression is important in BC pathogenesis by stimulating local invasion and the metastatic process, while its suppression is a valuable complementary therapeutic option [72,73,74,75,76,77].
(j) pH and environmental carcinogenesis: An integral explanation of human environmental carcinogenesis has been recently advanced, proposing that the oncogenic activity of many different carcinogens induce the same cancer-specific effects on cellular H^+^ dynamics (CPR). This recent and highly important integration into the H^+^-related paradigm strongly suggests the possibility of the existence of a universal mechanism responsible for environmental carcinogenesis [18,19].
(k) pH and cancer immunity: The acidic pHe of tumors has been considered to be the ultimate mechanism allowing them to escape from the anti-tumor immunity of the parasitized human organism. The final result is that this microenvironmental-intratumoral-extracellular (EC) low pHe creates a protective shield around cancer tissues with the onset of a state of anergy and immunosuppression mediated by the EC acidification-induced loss of function of T and NK cells. It also helps to explain the limitations and failures of checkpoint blockade in immunotherapy. Contrariwise, counteracting microenvironmental tumor acidity improves the antitumor responses to immunotherapy [20,21,47,78,79].
(l) pH and apoptosis: Malignant cell apoptosis is induced by pHi acidification while intracellular alkalization suppresses it [8,80,81,82,83].
(m) pH and drug resistance (MDR): Beyond the fact that the expression of P-glycoprotein (P-gp) leads to an elevation of pHi in cancer cells, an integral mechanism that explains MDR-based upon the selective changes in pHi and pHe (CPR), has been recently developed [84,85,86,87,88,89,90].
(n) pH, nanodrugs and liposomes: Systemic administration of nanoparticles disrupts microtubule dynamics and can be potentially useful for treatment on its own and in the overcoming of MDR. Some preparations of nanoparticles structure and delivery are highly pH-dependent [91,92,93].
(o) pH and aquaporins: Environmental pH changes either facilitate or hinder water diffusion across membranes while a rapid drop of cytosolic pH due to anoxia leads to closure of aquaporins in the plasma membrane [94,95].
(p) pH and autophagy: No matter that autophagy still appears to be a controversial issue, is considered a defensive survival mechanism of cancer cells in order to overcome drug-induced cellular stress and cytotoxicity. Acidic conditions increase autophagy in cancer cells suggesting that autophagy is a protective mechanism for tumor cells to survive under the microenvironmental acidic stress. Inhibiting autophagy may lead to a full cytotoxic effect [96,97,98,99,100] (for further details see the text).
(q) pH and repurposed drugs in cancer treatment: There is a wide array of intracellular acidifiers unrelated to PTIs or PPIs effects from many different origins and natures that have shown anticancer properties and in many cases are minimally toxic or not toxic at all [8,101,102,103].
(r) pH and photodynamic therapy in cancer: pHi acidification using photosensitizing agents leads to apoptosis and has been shown to suppress tumor growth and increase survival in animal tumors. Clinical studies in different solid tumors in humans are underway [104,105].
(s) pH and the spontaneous regression of cancer (SRC): The association of spontaneous regression of different cancers in humans with deep-seated systemic acid-base changes has been recognized for more than half a century [106,107,108,109,110,111,112].
(t) pH, evolution and cancer metabolism: From the fields of physics and chemistry to carcinogenesis, a pH-related reverse evolutionary process have been considered even from the times of Albert Szent-Györgyi (personal communication) [113,114,115,116,117].

## 3. All Phases of Breast Cancer Are Weaved into Each Other to Conform a Single, All-Comprehensive and Progressive, Multistage Unity

**Figure 1:**
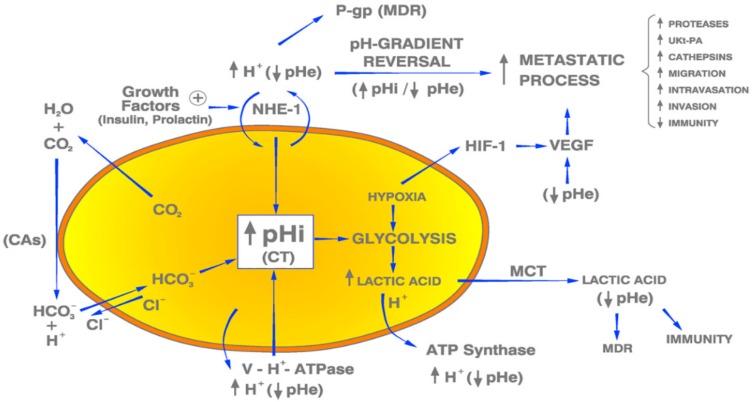
Proton transporters and proton pumps involved in the carcinogenicity of breast cancer and other malignant tumors. pHi: intracellular pH; CT: cell transformation; pHe: extracellular pH; PTs: proton transporters; PPs: Proton pumps; NHE-1: Na^+^/H^+^ antiporter; H^+^: hydrogen ion; CAs: carbonic anhydrases; MCT: monocarboxylate transporters; P-gp: P-glycoprotein; V-H^+^-ATPase: Vacuolar ATPase; MDR: Multiple drug resistance. Blue arrows: Induction. Black arrows: Result. Yellow color: intracellular space of a tumor cell.

All the above-mentioned oncological fields and subfields have in common a pivotal characteristic, namely, the aberrant pHi/pHe regulation of hydrogen (H^+^) ion dynamics [118], an abnormality that cannot be more opposed to the acid-base and energetic normality of non-cancerous cells and tissues. In a recent publication on brain malignancies, a large number of known etiopathogenic factors from many different natures and origins, all known to cause different human malignancies, were considered together [8,25]. Importantly, all the cancer-inducing factors listed in that review, but also in other previous reports of this group, act via a universal mediating mechanism, that is, an increase of cell pH secondary to the stimulation of the activity of NHE and/or other H^+^-related membrane-bound PTs and PPIs (Figure 1).

The nature and evolution of any solid tumor, and also of BC, makes it possible to accept that the deep-seated pH deregulations and/or highly disrupted H^+^-dynamics of malignancy are a fundamental factor behind a predetermined, progressive and staggered strategy of growth and dissemination (“the selective neostrategy of cancer cells and tissues”). This process begins with cell transformation and is closely followed by local growth and invasion under a highly hostile acid-base tumor microenvironment (TME). These initial phases are followed by neoangiogenesis, which favors metastatic dissemination and drug resistance. Each of these closely related periods of the natural evolution of cancer is not separated at all from the previous or subsequent ones but forms a Unitarian and dynamically active process that can even be considered a “conscious” and organized preprogrammed strategy. Despite this, the understanding raised by the pH-anticancer perspective has the potential to offer the possibility of applying therapeutic methods in a unique direction in all the phases of the malignant process, either in BC or in other solid tumors. This strategy is bound to interfere with each and all cancer hallmarks through different procedures acting from prevention to the treatment of advanced disease.

## 4. pH/NHE, Microenvironmental Acidosis and Immunity, Insulin, Prolactin, Estrogens, Genetic Abnormalities and Growth Factors in the Promotion of Breast Cancer

### 4.1. pH/NHE, H^+^ Extrusion and/or Intracellular Alkalization in the Etiology and Pathogenesis of Breast Cancer

In addition to NHE overexpression, H^+^ extrusion from cells can also be mediated by a cohort of other membrane-bound proton transporters, pumps and ion channels [8,15]. On one hand, these actors participate in keeping pHi normal to elevated, so preventing a low pHi-mediated therapeutic apoptosis. Such factors are carbonic anhydrases (CAs), mainly CAIX and CAXII, vacuolar H^+^-ATPase proton pumps, voltage gated sodium channels, sodium bicarbonate cotransporters, monocarboxylate transporters (MCTs), Cl^−^/HCO3^−^ exchangers and ATP-Synthase [15,48] (Figure 1). Different types of acid extruders like NHE1, NBCn1 and MCT4 are expressed in human mammary tumors, promoting growth of at least triple negative BC (TNBC) through synergistic and different mechanisms of action [56]. Paradoxically, the Na^+^/H^+^ exchanger regulatory factor 1 (NHERF1) presents a dual activity, either oncosuppressant or prooncogenic in invasive BC, depending of the cellular location of its activity [119]. Also, NHE1 and NBCn1 drive cell cycle progression in human BC cells, while their knocking down reduces proliferation and progression [70]. TME acidosis is also associated with pain in bone metastasis in BC [120]. Unfortunately, although one or more of these H^+^-extrusion systems substantially applies to all malignancies, a complete selective mapping of which PTs and PPs are overexpressed in each particular tumor is still missing. Therefore, at the moment the concerted use of all of them in pharmacological doses is recommended [53].

### 4.2. A Universal Mechanism as a Final Mediating Cause of Breast Cancer

It has been recently demonstrated that H^+^ efflux alone is sufficient to induce dysplasia and potentiate cancer growth and invasion by oncogenic Ras and that inhibiting H^+^ efflux induces cell death in invasive primary tumor mammary cells [38]. In the same line, the most striking results have been obtained by the group of Fliegel, showing that NHE-mediated H^+^ extrusion alone has a direct carcinogenic effect on breast cells [40]. The same alteration also plays a fundamental role in the metastatic process and in multiple drug resistance (MDR) [41,42,43,44]. In these studies, NHE1 hyperactivity and/or a high pHi act as an early and decisive driver in BC carcinogenesis and also in most, if not all, other human malignancies [37,121]. Moreover, the elevated pHi is also the main responsible actor for the secondary acidification of the extracellular/intratumoral microenvironment (TME). Importantly, H^+^ extrusion by itself has been implicated in the transition and progression from precancerous ductal carcinoma in situ to invasive BC. Of note, even the precancerous lesion already shows a higher than normal proton export rate [40,42,43]. Indeed, the invasive BC cells show a higher pHi and a higher production and exportation of H^+^ into the TME than noninvasive cells [40,53,122]. (Table 1). All these are qualitative and highly specific changes in the etiopathogenesis of BC.

### 4.3. Tumor Microenvironmental (TME) Acidosis and Immunity

There is a direct effect of tumor interstitial acidosis in hindering the antitumor immune response of the organism, another negative effect of the CPR. A complete review of the mechanisms by which tumor acidity disrupts the body immune defenses, locally and systemically, have been published by Huber et al. [20] (Table 1). These authors have shown how the acidity of the TME disrupts the immune defense mechanisms against malignant tumors, locally and systemically (Figure 1), enabling a relentless and uncontrolled tumor progression. Similar conclusions have been reached by other groups, relating aerobic glycolysis and lactic acid production with tumor invasion and even with MDR [126]. For all these reasons, the TME has been targeted by different methods in order to decrease, control and, if at all possible, revert, tumor extracellular acidity, both in animals and humans, in different malignant tumors. To this end, dietary lipids, PPIs or large daily amounts of sodium bicarbonate or other buffers have been used [51,158], occasionally with good results. The positive and antimetastatic effects of this strategy are secondary to the fact that acidity blocks T-cell activation and impairs tumor immunity [78]. Therapeutically, controlling TME acidity corrects T-cell dysfunction and allows to improve the efficacy of many other T-cell-based anticancer treatments [21,127,129]. A similar situation arises in lymph nodes, where activated T-cells are inhibited by acidosis [47]. The different methods to counteract TME acidity have been recently reviewed [22].

Most importantly, seminal research in this area by Marches et al. demonstrated that the anti-IgM-mediated induction of cell death in human B lymphoma cells is dependent on NHE1 inhibition and subsequent intracellular acidification. These findings do not appear to have been properly followed, in spite that they represent a synthesis of three different fields of modern oncology research: biochemistry, molecular biology and cancer immunity, all under one wide-ranged embracing unit [79].

### 4.4. Insulin (INS) and Breast Cancer

After all the experience accumulated on the carcinogenic effects of Na^+^/H^+^ overstimulation and/or an elevated pHi, it can be concluded that any factor that up-regulates this antiporter may have a carcinogenic activity on its target cells (Table 1). Through its stimulating effects on glycolysis INS is one of these metabolic factors [35,145]. INS presents a direct effect in raising pHi, which at the same time increases glycolysis, and probably these are the two reasons for its known tumor-stimulating properties [144]. This is reasonable too, since hyperinsulinemia and obesity have been associated with an increased incidence of BC [139,140] (Table 1).

Cancer cells are also associated with INS insensitivity (resistance), due to high oxidative stress, especially during malignant transformation, and this could be an earlier event of carcinogenesis [141]. Recently published data show that behind the effect of INS on resistance appears to be an abnormality of the pH/NHE-1 signaling pathway [142], with NHE-1 over-expression as the first known key event of transformation in carcinogenesis [23,37]. Moreover, microenvironmental acidification and even systemic metabolic acidosis in cancer are linked with INS resistance [159,160], both phenomena being a reflection of the metabolic complications of cancer, the latter in advanced and disseminated disease [161,162,163]. For these reasons, some antidiabetic drugs like sulfonylureas, known to act by stimulating the pancreatic secretion of insulin, may have a negative impact on cancer growth [164,165,166,167]. In contrast, other antidiabetic agents, like Rosiglitazone and Metformin, show promising anticancer properties as INS-sensitizing agents [168,169] (Table 2). From a clinical perspective, it has also been shown that the over-expression of INS or the IGF-1 gene is associated with a decrease in the life span of women with BC, while their deletion improves life span and may also decrease tumorigenesis [143,146,147]. Finally, a recently published and highly interesting contribution has shown that insulin resistance might be a secondary effect of an abnormal NHE-1 signaling pathway [142] (Table 1).

### 4.5. Prolactin and Breast Cancer

The role of prolactin (PRL) in the pathogenesis and progression of human BC is generally accepted [151]. Through NHE1 activation, this hormone stimulates growth, motility and invasiveness of BC, in this way contributing to the progression of the disease in a similar fashion that estrogens do [48,150] (Table 1). We agree with these authors that because of the effects of PRL, its inhibition should play a preventive, therapeutic and adjuvant role in the treatment of BC, as has already been suggested for other tumors [8].

Furthermore, there seems to exist a protumoral and synergistic interaction between PRL and growth hormone (GH) in stimulating the growth of certain tumors, BC among them [152]. In this vein, Clevenger et al. advanced that antagonists of PRL/PRL receptor interaction can be useful in the treatment of human BC, either alone or in combination with traditional antiestrogenic agents like tamoxifen and letrozole [151]. For all these reasons, PRL inhibitors such as bromocriptine or cabergolide (dopaminergic agonists drugs) should be taken into account as part of the armamentarium of repurposed drugs in BC therapy, even as a drug sensitizer [153].

### 4.6. Estrogens and Breast Cancer

Human BC is a heterogeneous disease classified in three major subtypes based on the expression of estrogen and progesterone receptors and human epidermal growth factor receptor-2 [256,257]. Among these BC subtypes, triple-negative BC results in a higher risk of metastatic dissemination and early death (Table 1).

Estrogens frequently play a crucial role in breast tumorigenesis by promoting cellular proliferation and decreasing apoptosis [154]. Interestingly, a recent study discussed why some tumors express ER^+^ (estrogen receptors) and not ER- [155]. One of the suggested explanations is that while ER^+^ tumor cells are highly vascularized ER- cells are better characterized by a higher expression of:

(a) NHE1 activity.
(b) Hypoxia-inducible Factor activity (HIF).
(c) Carbonic Anhydrases (CAs) activity: CA-XII expression relies on estradiol activity [156]. Therefore, ER^+^ is more likely to be associated with CA-XII rather than with CA-IX, while CA-IX is more frequently associated with ER- cells [212]. It has been shown that the selective inhibition of CA-XI improves the prognosis of the disease [200]. Although estrogens are growth factors, their effects or relations with the H^+^ dynamics of BC cells have not been well established. However, ER- BC cells seem to be associated with NHE1 activity [157] (Table 1). ERs show a high degree of heterogeneity in BC [156], as first reported by Puddefoot et al. in 1993 [258], and further confirmed by Leclercq some years later [259]. This heterogeneity implies, among other aspects, that at least four isoforms of ER alpha may exist, migrating to different isoelectric points in isoelectric focusing gels. Whether one or more of these different isoelectric points may be related or even contribute to changes in the pHi of mammary cells leading to BC remains to be established.

### 4.7. Ion Channels

It has been previously shown that ion channels are an important factor in the etiopathogenesis of cancer and neurodegenerative diseases, both pathologies staying at opposite ends of a pH-related metabolic spectrum [8]. It is also been demonstrated that NaV1.5-Na**^+^** channels are in a close association with NHE-1, both being overexpressed in BC, where they contribute together to degrade the tumoral microenvironment, stimulate the formation of invadopodia and foster the metastatic process in a similar manner that CPR does [54,74,75,76,149] (Table 1). Furthermore, ion channels are activated at low microenvironmental tumor pH in BC and other tumors, thus promoting cell proliferation and migration. In this context, ion channels become relevant therapeutic targets [148].

### 4.8. PTs, PPs and Growth Factors

NHE1-overexpression is stimulated by a myriad of factors, all of which induce a pathological and carcinogenic elevation of pHi as a response of cells of many different origins and locations [25] (Table 1). Hence, the possibility of a cause–effect relationship between *BRCA1* and *BRCA2* genetic mutations in BC and pH/NHE1 and/or other PTs upregulation, has been recently pointed out by these authors. Among hormones, growth factors and cytokines that have been shown to be protumoral, either in BC or in other solid tumors, apart from estrogens, human growth hormone (hGH), prolactin, insulin and EGF and its receptor, are VEGF, PDGF, certain interleukins and sex steroids, some of which up-regulate NHE1 (Figure 1) [8].

To this already extensive list, PPs should be added, as well as certain oncogenes, virus and gene products such as Bcl-2 [124], a dysfunctional p53 and many chemical products known to play a role in carcinogenesis [80,82]. Other carcinogenic NHE-related factors are chronic hypoxia and the hypoxia inducible factor (HIF) [260]. Even high glucose loads stimulate Na^+^/H^+^ activity [261].

It can be concluded that if so many unrelated etiopathogenic factors are known to be carcinogenic, the up-regulation of any of them, or several ones at the same time in a synergistic combination with other stimulators in the same direction, indicates that the pHi/pHe abnormalities exert their carcinogenic effect through the same acid-base intracellular (IC)/extracellular (EC), or pHi/pHe, deregulated dynamics. In addition, this suggests the existence of a universality of phenomenon involved in human carcinogenesis and cancer etiopathogenesis, BC being no exception to this rule [25].

### 4.9. NHE1-Related Genetics of Breast Cancer

The Na^+^/H^+^ exchanger isoform 1 (NHE1) is nowadays increasingly recognized as one of the most important factors involved in the etiology and pathogenesis of BC [38,40,41,42,43]. NHE1 has been found to be produced from the *APNH* gene located on chromosome 1p35-36, whose deletion has been blamed to be involved in the development of different tumors, BC among them [26]. Other genes have also been described to have a role in the genetic abnormalities behind BC metastasis [3]. These authors have screened 4200 target genes and discovered 133 and 113 migratory modulators of Hs578T and MDA-MB-231 cells, which are predictive of BC progression and bad prognosis. Other genetic mutations, like *BRCA1* and *BRCA2*, are known to be strongly associated with familial breast and ovarian cancers [262]. The possibility that these two genetic abnormalities can be dependent on NHE1 hyperactivity has recently been proposed. However, no factual evidence is available at the present time that can ascertain such cause–effect relationships [25]. Finally, other pathways known to be involved in the pathogenesis of BC seem to act via different mechanisms and linked to other genes [263]. It is important to realize, as we have previously suggested, that in order to exert their role on cellular metabolism, genetic aberrations do so through the mediation of the microenvironmental changes they secondarily induce, and not directly on their own [264].

Since there is no formal proof of a relationship between H^+^ -dynamics and *BRCA1* and *BRCA2* genetic abnormalities in BC, this important issue remains to be investigated. In the same line, it is most surprising the total lack of information relating inflammatory breast cancer (IBC) with PTs, PPs and H^+^-dynamics. Despite the known importance of TME in this aggressive kind of BC, the few reviews available on TME in IBC completely ignore such more than possible relationships or any other reference to the H^+^-related paradigm in BC and IBC etiopathogenesis [265].

## 5. Hydrogen Ion Dynamics in Multiple Drug Resistance (MDR) in Breast Cancer and Other Malignant Tumors: An Integral Approach to Its Etiopathogenesis and Mediating Mechanisms

Resistance of BC cells to drugs like doxorubicin (DOXO), paclitaxel and cis-platinum depends on pH regulation [89,125,179] (Table 2). DOXO resistance is related to a progressive increase of pHi, and could be suppressed by the addition of P-glycoprotein inhibitors like verapamil [204]. These findings, apart from showing the close relationship of P-gp and pHi, have made it possible to conclude that P-gp behaves as a proton (H^+^) extrusion pump [84].

Levels of the proton transporter (PT) NHE1 are significantly higher in BC and in resistant cancer cells when compared to adjacent normal tissues and selective cells [165]. A proton transport inhibitor (PTI) like cariporide (CP, HOE-642), induces apoptosis in MCF-7/ADR cells in vitro and in vivo and is associated with the intracellular accumulation of DOXO and G0/G1 cell cycle arrest. CP also induces tumor growth attenuation and diminishes tumor volume. This strongly suggests that NHE1 should be a promising adjuvant therapeutic target, not only in BC but also in a wide array of other malignant tumors [170]. Other PTs, like the HCO_3_^−^-cotransporter NBCn1 (Slc4a7), show similar effects, delaying BC development [58]. Finally, the association of PTIs with PPIs of the omeprazole family offers the possibility of further improving the effects of chemotherapy in metastatic BC [187], as well as in other malignant tumors [177]. Bringing all these findings together, it can be concluded that the association of PTIs and PPIs can have a synergistic effect in overcoming MDR in BC, apart from having a strong antitumoral effect on their own, from prevention to the treatment of advanced disease.

## 6. pH-Related Armamentarium in the Treatment of Breast Cancer

### 6.1. NHE Inhibitors and/or Intracellular Acidifiers

A fundamental therapeutic aim of the pH-related anticancer paradigm is directed to the concerted inhibition of NHE1 and other PTs and PPs in order to induce a progressively deep intracellular acidification (IA), which first would decrease glycolysis, then lead to tumor cell growth arrest, and finally induce selective apoptosis [54,176,266,267].

Amiloride (AM) was the first Na^+^/H^+^ antiporter introduced in medical oncology. However, AM is a weak and non-specific Na^+^/H^+^ antiporter inhibitor, but at least it is still commercially available as a diuretic (Table 2). Seminal research with AM showed a complete inhibition of lung metastasis of BC in animals. Such an effect was initially reported to be secondary to its inhibitory effects on urokinase-type plasminogen activator (UPa) [175]. AM has clearly shown antitumoral, antiangiogenic and antimetastatic effects [268,269]. Also, when decreasing pHi values with AM, VEGF mRNA expression and tumor growth are inhibited, at least in gastric and leukemic cells [17,172]. An occasional patient with advanced cancer has benefited for the chronic utilization of AM after mainstream chemotherapy had failed [171]. Finally, for a review on the positive effects of AM on basic cell behavior, see [118].

Among the more modern, potent and selective NHE1 inhibitors, cariporide (CP, HOE-642) induces apoptosis through lowering pHi [174]. CP also decreases angiogenesis and induces selective apoptosis mediated through Na**^+^**/H^+^ exchange inhibition [170]. Furthermore, CP induces apoptosis by overcoming paclitaxel resistance through NHE inhibition [180], and works synergistically with erlotinib in reducing metastasis in pancreatic cancer [208].

An even more potent NHE1 inhibitor than CP is the so called “Compound 9t” (C9t). C9t is five hundred times more potent against NHE1 than CP, apart from having a 1400-fold greater selectivity for NHE1 over NHE2 [178] (Table 2). Similarly, Phx-3, is a non-toxic NHE1 inhibitor that through selective apoptosis and intracellular acidification induces tumor growth regression after leukemic cell transplantation [32,182].

All available data indicate that these new and selective NHE1 inhibitors have a great potential as potent and selective anticancer agents in patients with different pathologies [170]. Additionally, it is difficult to understand why AM itself has not managed to find a place in bedside oncology, mainly in BC treatment, either as a preventive measure, as a complement of orthodox chemotherapy and/or as an antimetastatic factor. There can be two reasons for this: a) AM is not patentable, and b) in all areas of scientific research and medical life, sometimes the answers that are looked for in the future are waiting hiding in the past (Table 2).

### 6.2. Proton Pumps (PPs) and Their Inhibitors (PPIs) in Cancer and Breast Cancer

The over-expression of proton pump V-ATPases promote growth advantages to cancer cells of any origin, also disrupting pH homeostasis in the same CPR line of action [69,270]. Different publications have illuminated the crucial role of V-ATPases in tumor invasion and chemoresistance in several cancers, including BC. Therefore, as it happens with PTs inhibition, PPs inhibition of V-ATPases has recently become a novel therapeutic avenue for thwarting the highly abnormal H+ dynamics either in BC but also in other tumors of different origins and locations [15,49,194] (Table 2) Pretreatment with PPIs strongly enhances the in vitro efficacy of chemotherapeutic drugs against human BC cells and other malignancies [177,188,195,271,272]. Niikura showed that oral administration of a V-ATPase inhibitor to SCID mice carrying orthotropic BC xenografts resulted in delayed tumor growth and a decrease in bone metastasis [189]. Another in vitro report has shown the therapeutic effectiveness of PPIs in triple negative BC cell lines [69]. Finally, different preclinical studies support a direct anti-tumor effect of PPIs independently from cancer histology [190,191,192,273].

Two studies have been conducted in pets with spontaneous neoplasms using PPIs in combination with standard chemotherapy. The first one evaluated the ability of high dose lansoprazole to reverse chemoresistance in dogs and cats with cancers not responding to chemotherapy. In this study, the drug was used off-label with a three-day loading dosage of 5 mg/kg followed by a four-day maintenance regimen at 1 mg/kg as a chemosensitizer, combined with standard veterinary chemotherapy protocols. The results showed a reversal of chemoresistance in 23 out of 34 treated animals (67% response rate) (Table 2). A further study combined PPI with water alkalization and metronomic chemotherapy [184]. The cohort receiving alkalization showed enhanced tumor response, both in terms of the number of responders and the quality of response, when compared to the group receiving metronomic chemotherapy alone.

The application of this strategy to humans has led to the publication of two clinical trials. These studies evaluated the tolerability and effectiveness of high-dose PPIs in patients with osteosarcoma or metastatic BC [187,196]. One of these studies showed that the addition of PPIs to chemotherapy increased the effectiveness of chemotherapy in osteosarcoma patients. The second trial recruited women with metastatic BC that were randomized to receive either conventional chemotherapy or chemotherapy with alkalization [187]. In the latter study, patients receiving high dosage PPIs obtained the highest response rates and the longest survivals. Furthermore, there is a statistically significant survival advantage for women who continued their proton pump inhibition therapy (PPIT) after the completion of chemotherapy [271]. This in vivo data are also supported by in vitro investigations showing the effectiveness of PPIs in triple negative BC cell lines [69].

An indirect confirmation of the validity of PPIs and alkaline therapy as antitumor agents was provided by a published meta-analysis in head and neck tumor patients that found a better outcome for patients receiving PPIs [274]. A more recent study describes the outcome of a few patients with metastatic colorectal cancer who were refractory to standard chemotherapy and targeted therapies. The combination of high-dose rabeprazole (a PPI inhibitor) and metronomic capecitabine overcame drug resistance [275]. Despite of the very small number of patients studied, the association of rabeprazole and capecitabine resulted in long-lasting stable disease with good quality of life and relatively minor side effects.

More recent reports have shown that women affected by medical conditions suitable for PPIT treatment, e.g., peptic diseases, have a reduced risk for developing BC [67,194]. Both studies were performed in a very large cohort of patients with very convincing results. The data obtained also show that the beneficial effects of PPIT increases with age, the BC risk being reduced to a greater extent in older PPIT users, getting to 83.0% in the 50 to 64 years old cohort. These data are of particular importance in women with a higher risk of developing BC, like those with a family history of BC, as well as women treated with long term hormone replacement therapy during and after menopause. These studies also support the results obtained in women with metastatic BC, particularly those with triple negative BC when exclusively treated with high dosage PPIs, while intermittent high dose PPIs also enhances the antitumor effects of chemotherapy in metastatic BC [187]. Another three articles recently suggested reconsidering the use of PPIs in cancer therapy [186,276,277]. Their conclusions are highly convincing and important for the treatment of a disease like BC that is becoming an increasing killer worldwide.

Finally, since PPIs are prodrugs requiring activation in the acidic microenvironment of solid tumors, they appear particularly suitable to be used as anticancer drugs in the very acidic tumor microenvironment [22], this obviously being the case of BC as well. Moreover, while it is not entirely scientifically supported, it appears conceivable that PPIs may also affect the systemic pH by buffering the stomach, that is their main target [185].

### 6.3. Melatonin (MT) in Breast Cancer

Melatonin (MT) has been shown to function as an antiestrogenic agent, and only for this reason should be strongly considered for BC treatment [215,219] (Table 2). Sonehara et al. showed that treatment with MT modulates tumor aggressiveness, increasing apoptosis under microenvironmental acidosis in BC cell lines [220]. Importantly, MT has been shown to be a significant antiangiogenic agent by downregulating VEGF expression in human BC cells [214,221,225]. Previous studies have also indicated that MT suppresses tumor aerobic metabolism (Warburg effect) and cell-signaling pathways that are key for the proliferation and survival of BC cells as well as for metastasis and resistance to anti-cancer drugs [215,222,223,278]. MT treatment of breast tumor cells decreases the HIF-1α gene, HIF-1α expression and regulates glucose metabolism as well as the expression of protumoral factors like GLUT1, GLUT3, CA-IX and CA-XII, indicating that MT controls hypoxia and tumor progression [279].

Otherwise, traditional oncology has clearly shown that antiestrogens are an effective measure in the treatment of ER + BC, using either Tamoxifen or Letrozole. In this vein, MT, as well as resveratrol, also appears to function as aromatase inhibitors, so becoming further candidates in the adjuvant treatment of ER + BC [226]. In addition to these mechanisms, MT shows many other anticancer and oncostatic effects in BC [216]. Among them, MT enhances the sensitivity to classical anti-cancer agents [215].

Interestingly, a recent in vitro study provides evidence about the positive effects of a novel MT-TMX drug combination in the treatment of BC. It is known that TMX use may eventually lead to resistance. However, this seems to be overcome by the novel MT-TMX conjugates [217]. Furthermore, an inverse correlation between nocturnal melatonin levels and the development of BC has been confirmed [213,219]. This appears to be related to the loss of the day/night MT circadian rhythm, increasing the risk of BC development in female night workers. In fact, it has been shown that women with BC had lower plasma levels of MT than normal women, and these levels are even lower in nurses working shifts [218].

In summary, it is beyond doubt that MT plays an important role in the prevention and treatment of BC. Although its primary effect seems to be exerted at a mitochondrial level by regulating aerobic metabolism, MT also decreases angiogenesis and proliferation while promoting apoptosis [214,215,220,221,224].

### 6.4. Cisplatin (CDDP) and pH/NHE in Breast Cancer

Cisplatin (CDDP) has been used in the treatment of BC and other malignant tumors for a long time, and it continues to be widely used nowadays [205,206,280]. From its first introduction in bedside oncology, different and unrelated mechanisms of action for CDDP have been described [210,281]. Most importantly, an almost completely disregarded issue has been the fact that CDDP can significantly modify the intracellular pH of cancer cells by inducing cytoplasmatic acidification through a CDDP-mediated inhibition of H^+^ extrusion secondary to downregulation of NHE-1 [206,209,210,211] (Table 2). Most recently, this has been considered the first effect of CDDP [209]. Contrariwise, the activity of NHE-1 and its effect on elevating pHi increases CDDP resistance to treatment [209]. Apart from inducing pHi acidification, CDDP shifts cervical cancer cells from glycolysis to oxidative metabolism, and this is accompanied by inhibition of cancer cell growth. In these studies, cancer cells either recover, maintaining an alkaline pHi to survive and proliferate, although at reduced growth rates, or die [282].

### 6.5. pH and MDR in Breast Cancer: An Integrated Approach to Treatment

For many years, P-glycoprotein (P-gp) has been held to be the main responsible mechanism for multidrug resistance (MDR) in solid tumors. However, seminal research in this area initially showed that a progressive increase in pHi was correlated with the level of doxorubicin (DOXO) resistance in human lung tumor cells. In this case, drug resistance was counteracted upon the addition of verapamil, an inhibitor of P-gp activity [204]. At that time, the fact that P-gp affected pHi was already suggested. Furthermore, during the last two decades pH alterations have been shown to be behind the most fundamental aspects of MDR [84,90,130,283,284]. Indeed, it has been recently shown that P-gp needs a pH gradient in order to function [86,285] and an integrated mechanism to explain MDR has been developed based upon the H^+^-dynamics of the microenvironment of tumors (CPR) [49,84,86,89,130,131,179,286]. This new and integral model demonstrates that in MDR the CPR of cancer cells and tissues and the P-gp expression are related in a direct cause-effect relationship and cannot be separated from each other.

This integral approach to MDR has proposed that the therapeutic failure to induce cytoplasmic acidification and/or reverse CPR is the main underlying factor for drug resistance, which suggests that MDR and resistance to the induction of the low pHi-mediated therapeutic apoptosis are also one and the same thing. Furthermore, the expression of P-gp leads to an elevation of pHi [87,287], while intracellular acidification down-regulates the MDR transporter [87,288,289]. Finally, extracellular acidification increases the activity of P-gp, inducing MDR in different cancer cells and tissues [49,130,134]. In summary, all these findings perfectly fit into each other, meaning that the therapeutic induction of a reversion of CPR is also the key and fundamental target in overcoming MDR, probably in all malignant tumors, like it is in pH-related cancer treatment. This is in line with all the other integrations made possible when approached through the all-comprehensive pH-centric anticancer paradigm [8,24,133].

### 6.6. Repurposed Drugs in Breast Cancer Treatment

TME acidification makes BC more aggressive [135,290]. This intratumoral but extracellular acidosis, mainly caused by lactic acid production, is related to an increased aerobic glycolysis (Warburg effect) (Figure 1), being fundamental in promoting invasiveness of BC cells [136]. On the contrary, chronic administration of sodium bicarbonate to nude mice implanted with human BC reduces the number and size of metastases [51] (Table 2).

Among the many mechanisms responsible for the regulation of the protoplasmic acid-base balance (Carbonic anhydrases (CAs), Monocarboxylate Transporters (MCT), ATP synthase, V-ATPases and Na**^+^**/H^+^ exchanger isoform 1(NHE1), CAIX appears to be a critical mediator for the expansion of BC in hypoxic niches, sustaining the mesenchymal and ‘stemness’ phenotypes of these cells [202]. CAIX activity affects the uptake and toxicity of anticancer drugs and is associated with a bad prognosis. Also, Erb-2 expression is associated with bad prognosis [201]. The CA inhibitor Acetazolamide (AZM) enhances DOXO toxicity but reduces Melphalan toxicity in BC cell lines that express CAIX, which is also a target for BC anticancer treatment [101]. In hypoxic BC tumor cells, the inhibition of different H+-extruding mechanisms has been proposed as a therapeutic strategy, while among them CAIX is considered to represent the most promising target [203].

Furthermore, V-ATPase and MCT4 are both major microenvironmental acidifying mechanisms in human BC cell lines [137]. Indeed, MCTs are often upregulated in BC tissue [197], while MCT4 is a clear therapeutic target, at least in certain subtypes of BC [59]. Thus, it is logical that targeting lactate transport with MCT inhibitors such as Quercetin suppresses BC growth and improves tumor immune response [102,181,198,291]. Other MCT inhibitors such as Simvastatin and Phloretin have also been found to be active against BC cells [103,227] (Table 2).

Lonidamine was first introduced in 1979 as an antispermatogenic agent. It inhibits L-lactate transport through activity on MCT1, MCT2 and MCT4, causing selective intracellular acidification of tumors. It has been active in metastatic BC patients, but is not commercialized any more [230].

The n-3 polyunsaturated Docosahexaenoic acid (DHA, 22:6n-3), is effective in increasing survival and chemotherapy efficacy in BC patients with metastasis [231], inhibiting NaV1.5 current and NHE-1 activity in human BC cells [232]. The daily doses used in the clinical trials were in the range of 1,800 mg DHA/day, while a single case report showed a positive result in a BC patient that only used 480 mg DHA/day as part of a more extensive supplementary cocktail using a multitargeted approach [228].

Drug screening has identified an FDA approved drug, Niclosamide as an inhibitor of BC stem-like cells [233]. Another group of compounds known to induce cytosolic acidification are the K+ ionophores. These compounds promote the outflow of K^+^ from the mitochondria as well as from the cytoplasm, mediating an H^+^/K^+^ exchange across lipid membranes. The result is the induction of an intracellular accumulation of protons [8]. One such K^+^ ionophore is the antibiotic Salinomycin. Promising results from a few clinical pilot studies indicate that Salinomycin is able to induce partial clinical regression of heavily pretreated and therapy-resistant cancers, including BC [234] (Table 2). Finally, for a more complete exposure of repurposed drugs in preclinical and clinical oncology, see [8].

### 6.7. Metformin (MET) in Breast Cancer

Boosting glycolysis with mitochondria inhibitors such as Metformin (MET) have been proposed as a method to decrease pHi in various cancer cell lines, BC among them [52], alone and/or in combination with the MCT inhibitor Simvastatin [53] (Table 2). MET has been considered to be a viable anticancer drug since it induces intracellular hyperacidification in tumor xenograft models through inhibition of Wnt signaling, a feature found to be selective for cancer cells [52]. MET was introduced as an anticancer agent in clinical oncology after it was reported to decrease mortality of BC patients [235,236,245,246,247], increasing the survival of triple-negative BC patients [247]. MET can act as an anticancer drug through its activity on several glucose transporters [292] known to be associated with BC [237,238,293,294]. Some of the anticancer-related effects of MET are:

(i) It reduces circulating insulin and insulin/IGF-1 receptor-mediated activation of the PI3K pathway [245].
(ii) MET inhibits the expression of the Hypoxia Inducible Factor 1 alpha (HIF-1α) gene expression, increases Pyruvate Dehydrogenase (PDH) gene expression [249] and decreases Warburg metabolism [35]. Additionally, HIF-1α is fundamental in tumoral angiogenesis and induces the expression of VEGF in BC [134,252,260,295]. Through this and other mechanisms MET also inhibits cancer growth, including triple-negative BC (TNBC) [254]. VC REMOVE:
(iii) As an inhibitor of tumor angiogenesis [239,248,250,251], a recent study revealed the impact of MET inhibitory effect on microvasculature [296]. Through this antiangiogenic effect MET can also improve tumor prognosis [155,252].
(iv) MET can reduce tumor progression through AMPK inactivation [297,298], although the opposite effect has also been reported [299].
(v) MET can inhibit cancer migration, invasion and metastasis in BC and other tumors [240,300,301,302,303].
(vi) MET is also active via the inhibition of the hedgehog signaling pathway in tumors like BC [241,242].

Finally, MET has not only been used as an anticancer agent by itself, but is also useful as an adjuvant to other cancer chemotherapy agents, particularly being able to reduce the side-effects of DOXO [304]. Moreover, MET has been reported to act synergistically with chemotherapy, also decreasing its dosages [243], and has even been used to target resistant cells in BC and other tumors [244]. MET has even been considered to be a radio-sensitizer agent [255].

### 6.8. Autophagy and Cannibalism in Breast Cancer

Autophagy; however still a controversial issue, has been shown to represent an adaptive survival mechanism to overcome drug-induced cellular stress and cytotoxicity. This was demonstrated using PPIs, which induced early accumulation of autophagosomes, also reducing the autophagic flux. Notably, the inhibition of autophagy by knockdown of Atg5 and Beclin-1 expression significantly increased PPIs cytotoxicity [305], suggesting that autophagy may exert a protective role in cancer cells treated with PPIs, and that inhibiting the autophagic process may lead to higher cytotoxic effects and improve therapeutic efficacy. Furthermore, TME acidic conditions increase autophagy in cancer, suggesting that it may protect tumor cells allowing them to survive under microenvironmental acidic stress [96]. This was shown in BC cell lines [97], suggesting that PPIs can help in improving anticancer therapeutic efficacy in a broad spectrum of cancers.

In the same line, Salinomycin exerts a potent inhibition activity of the autophagic flux, mainly when cells are cultured in acidic conditions. This has been shown in tumor cells obtained from BC patients [98]. Functioning as a potassium ionophore, Salinomycin has been shown to contribute in further inhibiting lysosomal degradation, supporting the idea of using a broad panel of other ion inhibitors in modulating autophagy in cancer [306]. From these studies it appears that H^+^/K^+^ ATPases or V-ATPases may represent preferential targets to inhibit autophagy. However, from other studies it appears that the mechanism malignant tumor cells use to face off nutrient starvation, in acidic conditions as well, is cannibalism [307,308]. Cannibalism has been shown in BC as well [309], implying that autophagy may exert a different role in cancer cells than a mechanism of self-feeding in nutrient low supply conditions, inasmuch BC cells feed on other cells that within the tumor are in fact siblings cells, i.e., cells from the same individual. This implies the existence of an entirely new phenomenon than can be called self-cannibalism, or even auto-cannibalism, and that may be considered a specific characteristic of malignant tumors [308]. In fact, it has been shown that self-cannibalism is a feature of cells mainly derived from metastatic lesions rather than from the primary tumor [307].

## 7. Conclusions

If many unrelated etiopathogenic factors of so many different natures and origins cause cancer, the upregulation of any or several of them, alone or in a synergistic combination with other stimulators of cellular hydrogen ion (H^+^) extrusion, indicates that the pHi/pHe abnormalities selectively induced in cancer cells and tissues mediate in the behavior of malignant tumors like breast cancer in all its phases of development. This also suggests the existence of a universality of phenomenon involved in the carcinogenesis of breast cancer and other human tumors. In this contribution, the multifactorial etiological and etiopathogenetic factors in breast cancer are considered all together, which allows to propose an integrated and unidirectional approach to the therapeutics of this deadly disease. It is also shown that all the areas and hallmarks of cancer research, perhaps with the exception of radiotherapy, can be integrated under the same Unitarian acid-base paradigm. Interestingly, there is a surprising lack of information relating to the pH/NHE-related paradigm and genetic abnormalities like *BRCA1* and *BRCA2* positive tumors and of H^+^-dynamics with inflammatory breast cancer, despite the known importance of tumor microenvironment in this aggressive form of the disease.

The ultimate goal of this integral approach to malignancy is to target the selective molecular and metabolic-dependent acid-base disruptions specific to cancer cells using different and concerted synergistic methods. The final aim of therapy is to take advantage of the H^+^-related selective abnormalities that cancer cells and tissues possess over their normal counterparts, in order to exploit such differences in treatment. It is concluded that any attempts to induce a low pHi-mediated apoptosis can be the cancer-specific and fundamental strategy to treat breast cancer as well as other human malignant tumors. The pH-centric anticancer paradigm recognizes that any attempts to selectively induce an intracellular hyperacidification incompatible with the life of cancer cells, and/or reverting cancer proton reversal (CPR), are its main therapeutic targets. The pending issue nowadays is to find that old Ehrlich′s magic bullet that can selectively achieve that, if such a weapon exists. Otherwise, the concerted utilization of some of the measures conceptualized and described here, is likely to become a useful and integrated alternative, both nowadays and in the near future, to more efficiently treat all forms of breast cancer.

## Figures and Tables

**Table 1:** pH-related and -unrelated mechanisms in the etiopathogenesis and progression of breast cancer. BC: Breast cancer; TME: Tumor microenvironment; MDR: Multidrug resistance; NHE: Na^+^/H^+^ antiporter: CAs: Carbonic anhydrases; ER-: Estrogen negative cells; PTIs: Proton transport inhibitors; PPIs; Proton pump inhibitors.

Mechanisms	Summary	References
H^+^ extrusion and/or elevated pHi	H^+^ extrusion from cells is sufficient to induce transformation, growth and invasion in BC and other tumors. NHE-mediated H^+^ extrusion by itself has a carcinogenic effect on breast cells and increases MDR.	[8,23,25,37,38,39,40,41,42,43,44,48,54,59,122,123,124,125]
Tumor micro-environmental (TME) acidosis, immunity and MDR	Acidity of the TME disrupts the body immune defense mechanisms towards malignant tumors, locally and systemically. This allows a relentless and uncontrolled tumor progression. TME also has an essential role in the progression of inflammatory BC. Thus, TME is a novel therapeutic target in BC. TME acidity also induces MDR.	[20,21,22,47,49,50,51,78,79,84,87,116,120,126,127,128,129,130,131,132,133,134,135,136,137,138]
Insulin (INS) and insulin-like growth factor-1 (IGF-1)	INS and INS-resistance have a direct effect in raising pHi and are associated with breast cancer carcinogenicity and progression. Over-expression of insulin/insulin-like growth factor-1 is associated with a decrease in the life span of women with BC.	[3,35,39,139,140,141,142,143,144,145,146,147,148,149]
Prolactin (PRL)	PRL stimulates growth, motility and invasiveness of BC cells through NHE1 activation. In this way contributes to the metastatic process of human BC and becomes another therapeutic target.	[150,151,152,153]
Estrogens	Estrogens play a crucial role in breast tumorigenesis by promoting cell proliferation and decreasing apoptosis. ER-cells are considered to have a higher expression of NHE activity and are preferably associated with CA-IX over CA-XII. Inhibition of CA-IX improves the prognosis of the disease.	[4,154,155,156,157]
Ion channels	NaV1.5-Na^+^channels associated with NHE-1 are overexpressed in BC, stimulating the formation of invadopodia, facilitating local growth and the metastatic process.	[8,72,73,75,76,77,148]
PTs, PPs, and Growth factors	NHE1-overexpression is stimulated by a myriad of factors, which alone or in combination induce a carcinogenic elevation of pHi as the oncogenic response of normal cells of different origins and locations. Carbonic anhydrases (CAs) also have an important role in the pathogenesis of BC, mainly in hypoxic conditions. NHE1 levels are significantly higher in BC tissue than in adjacent normal tissue, and also in resistant BC cells when compared to sensitive cells.	[8,25,41,55,56,57,58,59,60,61,62,63,64,65,66,67,68,69,70,71]

**Table 2:** pH-related drugs with present and potential benefit in the treatment of breast cancer. BC: Breast cancer; NHE: Na^+^/H^+^ antiporter; MDR: Multiple drug resistance; P-gp: P-glycoprotein; CAs: Carbonic anhydrases; ER^+^: Estrogen positive cells; ER-: Estrogen negative cells; PTIs: Proton transport inhibitors; PPIs; Proton pump inhibitors. TME: tumor microenvironment.

Drug	Summary	References
Amiloride (AM) (and/or liposomal amiloride), proton transport inhibitors (PTIs) and intracellular (IC) acidifiers	AM is a non-specific NHE inhibitor first introduced for human use as a K^+^ sparing diuretic. It works as an antiangiogenic agent and has proved to be most effective as an antimetastatic drug in transplanted breast tumors in animals. A positive clinical experience in an occasional patient has been reported with its chronic use when traditional chemotherapy had failed. Also, the many anti-cancer effects of AM have been fully described. However, its utilization has not entered clinical trials in bedside oncology. (For further details, see the text).	[8,16,25,26,32,53,54,60,63,64,67,68,69,79,82,88,101,110,118,129,158,165,170,171,172,173,174,175,176,177,178,179,180,181,182,183]
Proton pump inhibitors (PPIs) and TME alkalization	PPIs are useful in the prevention of BC. Besides, the clinical utilization of V-ATPase inhibitors is a novel therapeutic measure to counteract the abnormal proton dynamics of BC and other tumors. PPIs also benefit from the microenvironmental acidity of tumors. Preclinical and clinical studies also support a direct anti-tumor effect of PPIs in BC and other solid tumors.	[22,49,51,67,78,85,128,130,177,184,185,186,187,188,189,190,191,192,193,194,195,196]
Monocarboxylate transport (MCT) inhibitors	Quercetin is a pan-monocarboxylate transporter (MCT) inhibitor and intracellular acidifier. Liposomal quercetin is also available, since gastrointestinal absorption is very limited in the non-liposomal drug form.	[101,102,137,197,198,199]
Acetazolamide (AZM)	AZM is a carbonic anhydrase (CA) pan-inhibitor and cell acidifier. CAIX inhibition significantly reduces invasion of BC cells and represents a most promising drug in the treatment of BC, alone or in combination with different NHE inhibitors.	[63,65,165,183,200,201,202,203]
Doxorubicin (DOXO)	There is a progressive increase in resistance to DOXO by increasing elevations of pHi, resistance that is suppressed by P-gp inhibitors, while P-gp also increases pHi. MDR is characterized by a reversal of the pH gradient (cancer proton reversal or CPR) across cancer cell membranes.	[8,25,84,88,89,90,101,179,204]
Paclitaxel	The inhibition of NHE1, which is fundamental in the chemotherapy of triple-negative BC metastasis, improves the efficacy of Paclitaxel and mediates in Paclitaxel-induced apoptosis of BC cells.	[40,42,44,179,180,205,206,207,208]
Cis-platinum (CDDP)	The first effect of CDDP is the induction of intracellular acidification by inhibiting H+ extrusion secondary to downregulation of NHE-1. Contrariwise, the activity of NHE-1 and its effect on elevating pHi increases CDDP resistance to treatment.	[206,209,210,211]
Antiestrogens	ER- breast cancer cells have a higher expression of NHE activity and are preferably associated with CA-IX over CA-XII. Inhibition of CA-IX improves the prognosis of the disease. Although the role of Tamoxifen and Letrozole is well established, no further connections among pH dynamics and these antiestrogens have been described.	[4,5,154,155,156,157,200,212,213]
Melatonin (MT)	MT has an antiestrogenic effect and only for this reason it should be contemplated in BC therapy. Treatment with MT modulates tumor aggressiveness and increases apoptosis n BC cell lines. MT also suppresses tumor aerobic metabolism (the Warburg effect) and decreases breast cancer angiogenesis and metastasis.	[214,215,216,217,218,219,220,221,222,223,224,225]
Cariporide (CP)	CP (HOE-642) is a powerful NHE1 inhibitor that, unfortunately, is not available for clinical use in bedside oncology. It induces apoptotic cells death in different malignant tumors.	[8,25,35,170]
Compound 9t (C9t) (Unavailable)	C9t is 500-fold more potent against NHE1 than cariporide and has a 1400-fold greater selectivity for NHE1 over NHE2. Besides, C9t is orally bioavailable, has low side-effects in mice and it presents a significantly improved safety profile over other NHE1inhibitors.	[8,35,178]
Phx-3	Phx-3 is a potent, selective and non-toxic NHE1 inhibitor that triggers apoptosis in a variety of cancer cell lines and is highly effective in some animal tumor models.	[8,32,182]
Repurposed drugs	Phloretin, Lonidamine, Niclosamide, Docosaexaenoic acid, Salinomycin and Simvastatin have been reported to be useful in the treatment of BC because of their pH-related effects. Resveratrol also has a role as an aromatase inhibitor. (For further details, see the text).	[8,103,226,227,228,229,230,231,232,233,234]
Metformin (MET)	MET has been introduced as an anticancer agent in BC. It induces intracellular hyperacidification in tumor xenograft models. MET has been reported to inhibit insulin and insulin/IGF-1, HIF-1α, Warburg metabolism, gene expression, angiogenesis, cancer migration, invasion and metastasis, apart from reducing the side effects of doxorubicin. MET has also been reported to act synergistically with chemotherapy and decrease its dosages, thus, its side-effects. It has also been used to target resistant cells in BC and has been considered a radio-sensitizer.	[235,236,237,238,239,240,241,242,243,244,245,246,247,248,249,250,251,252,253,254,255]
